# Case-oriented pathways analysis in pancreatic adenocarcinoma using data from a sleeping beauty transposon mutagenesis screen

**DOI:** 10.1186/s12920-016-0176-7

**Published:** 2016-04-01

**Authors:** Yen-Yi Ho, Timothy K. Starr, Rebecca S. LaRue, David A. Largaespada

**Affiliations:** 1 Division of Biostatistics, School of Public Health, University of Minnesota, 420 Delaware St. SE, Minneapolis, Minnesota USA; 2 Masonic Cancer Center, University of Minnesota, 425 E River Pkwy, Minneapolis, Minnesota USA; 3 Department of Pediatrics, 2231 6th St. SE, Minneapolis, MN 55455 Minnesota USA; 4 Department of Obstetrics, Gynecology & Women’s Health, MMC 395, 420 Delaware St SE, Minneapolis, MN 55455 Minnesota USA; 5 Minnesota Super Computing Institute, 599 Walter Library, 117 Pleasant Street SE, Minneapolis, MN 55455 Minnesota USA

**Keywords:** Forward genetic screen, *Sleeping Beauty transposon*, Case-oriented gene set analysis, Pathways correlations, CIS, Common insertion sites

## Abstract

**Background:**

Mutation studies of pancreatic ductal adenocarcinoma (PDA) have revealed complicated heterogeneous genomic landscapes of the disease. These studies cataloged a number of genes mutated at high frequencies, but also report a very large number of genes mutated in lower percentages of tumors. Taking advantage of a well-established forward genetic screening technique, with the Sleeping Beauty (SB) transposon, several studies produced PDA and discovered a number of common insertion sites (CIS) and associated genes that are recurrently mutated at high frequencies. As with human mutation studies, a very large number of genes were found to be altered by transposon insertion at low frequencies. These low frequency CIS associated genes may be very valuable to consider for their roles in cancer, since collectively they might emerge from a core group of genetic pathways.

**Result:**

In this paper, we determined whether the genetic mutations in SB-accelerated PDA occur within a collated group of biological processes defined as gene sets. The approach considered both genes mutated in high and lower frequencies. We implemented a case-oriented, gene set enrichment analysis (CO-GSEA) on SB altered genes in PDA. Compared to traditional GSEA, CO-GSEA enables us to consider individual characteristics of mutation profiles of each PDA tumor. We identified genetic pathways with higher numbers of genetic mutations than expected by chance. We also present the correlations between these significant enriched genetic pathways, and their associations with CIS genes.

**Conclusion:**

These data suggest that certain pathway alterations cooperate in PDA development.

**Electronic supplementary material:**

The online version of this article (doi:10.1186/s12920-016-0176-7) contains supplementary material, which is available to authorized users.

## Background

The molecular analysis of human cancer cells has revealed a startling amount of genetic and epigenetic heterogeneity. In recent years, forward genetic screens have taken place in mice using DNA transposons, primarily Sleeping Beauty (SB) [1]. The SB-based approach has been successfully employed to induce many different forms of cancer such as brain tumors, sarcomas, hematopoietic malignancies, and carcinomas [2, 3] via insertional mutagenesis. A large number of of loci recurrently mutated by insertion of SB transposons called common insertion sites (CIS) have been identified [4]. The general impression from these studies is one of tremendous genetic complexity.

Recent large-scale analyses of human cancer genomes mirrors these results in general. Most types of human cancer harbor a small number of genes that are altered in a high percentage of cases, so called “mountains”, and a large number of genes altered in a low percentage of cases, so called “hills”. In addition, two patients diagnosed with the same type of cancer often show distinct genetic alternations, however, the disrupted pathways tend to be similar among patients [5].

Conventional pathway analysis approaches usually obtain gene-based scores by summarizing data across tumor cases, then calculating pathway statistics using the scores of the genes in the pathway. However, these approaches could potentially lose information regarding whether multiple mutations in a pathway are from a single patient or multiple cases with a single mutation at various genes in the pathway. In contrast, case-oriented gene set analysis (CO-GSEA) can consider the two situations differently and hence can incorporate heterogeneity of each tumor case into the analysis. This approach provides a case-based score for each pathway and further enhances the study of correlation of mutation events between pathways, as well as between genes and pathways. It has recently been applied to the analysis of human tumors [6].

Pancreatic ductal adenocarcinoma (PDA) is the fourth leading cause of death due to cancer, with over a 98 % case-fatality rate. The crucial molecular events, required for progression from a pre-invasive and non-life threatening state to an invasive and metastatic lethal condition, are not well-understood. We previously reported the results of a SB transposon-based forward genetic screen for drivers of PDA in mice expressing the Kras^G12D^ oncogene in epithelial cells of the pancreas [7]. Our screen revealed new candidate genes for PDA and confirmed the importance of many genes and pathways previously implicated in human PDA. The most commonly mutated gene was the X chromosome-linked deubiquitinase *Usp9x*, which was inactivated in over 50 % of the tumors. In addition, several hundred candidate PDA genes were identified as CIS in this screen.

In this paper, we report analyses intended to determine whether a core group biological processes or pathways are populated by genes from CIS. We applied a less stringent criterion to consider CIS associated genes that mutated both at high frequencies (mountains) and at lower frequencies (hills). Secondly, we determined whether non-random associations between alteration of genes in certain pathways or biological pathways exist by analysis of CIS from individual tumors.

## Results and discussion

### Certain pathways are enriched in CIS-associated genes

We collected insertional mutatgenesis data of tumor samples from 146 *Kras*^*LSL-G12D*^*; Pdx1-cre; T2/Onc; Rosa26-LSL-SB13* mice. To determine whether a core group of pathways were enriched with CIS-associated genes than reported previously [7], we analyszed 968 CIS with uncorrected *p* value <10^−4^ from TapDance. Among these, 239 genes were mapped an grouped into 281 KEGG curated pathways categories. After excluding pathways with less than 6 genes, 272 KEGG pathways remain in the following analysis.

**Fig. 1:**
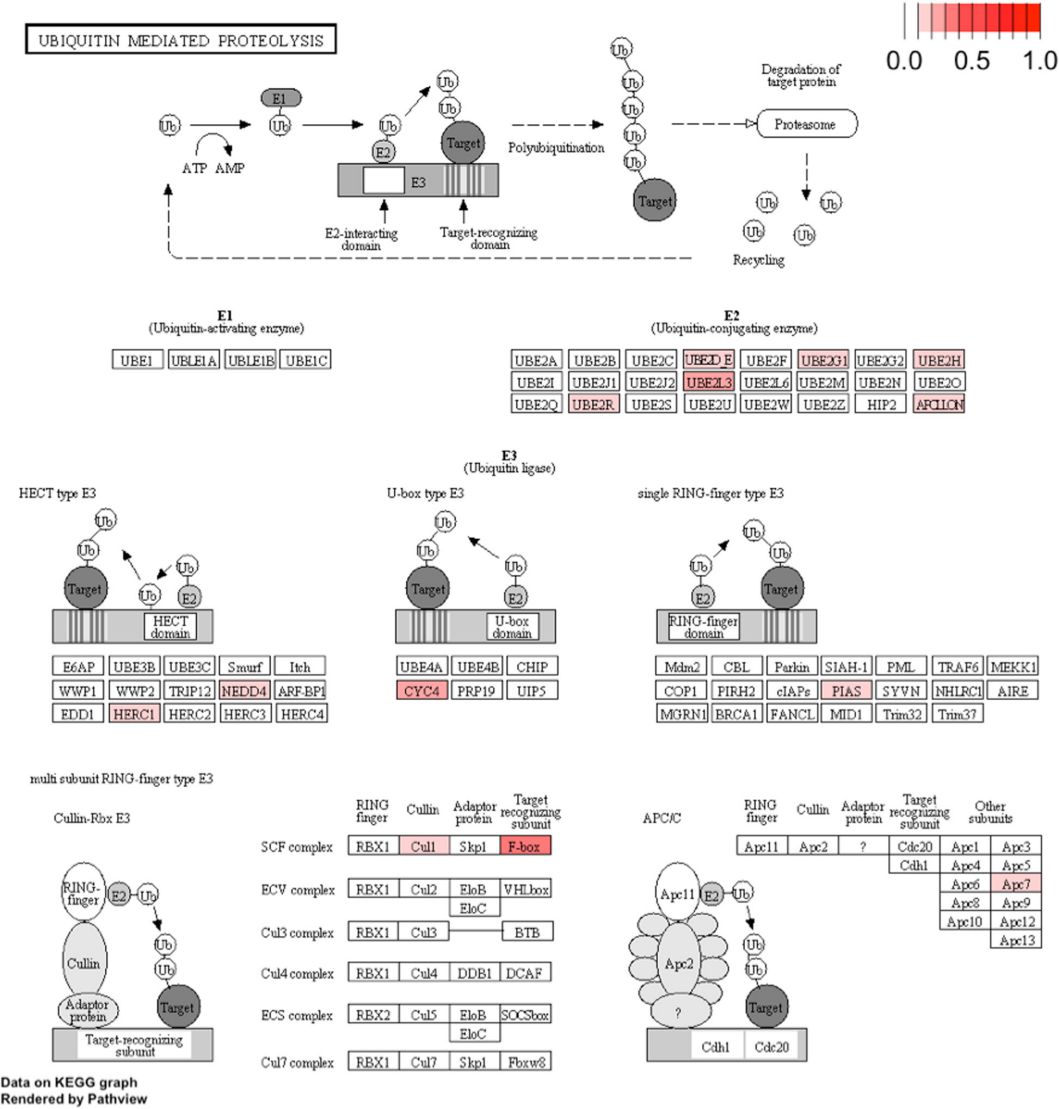
Frequently mutated genes in ubiquitin mediated proteolysis pathway. Darker red color indicates higher mutation frequencies in mice

**Fig. 2:**
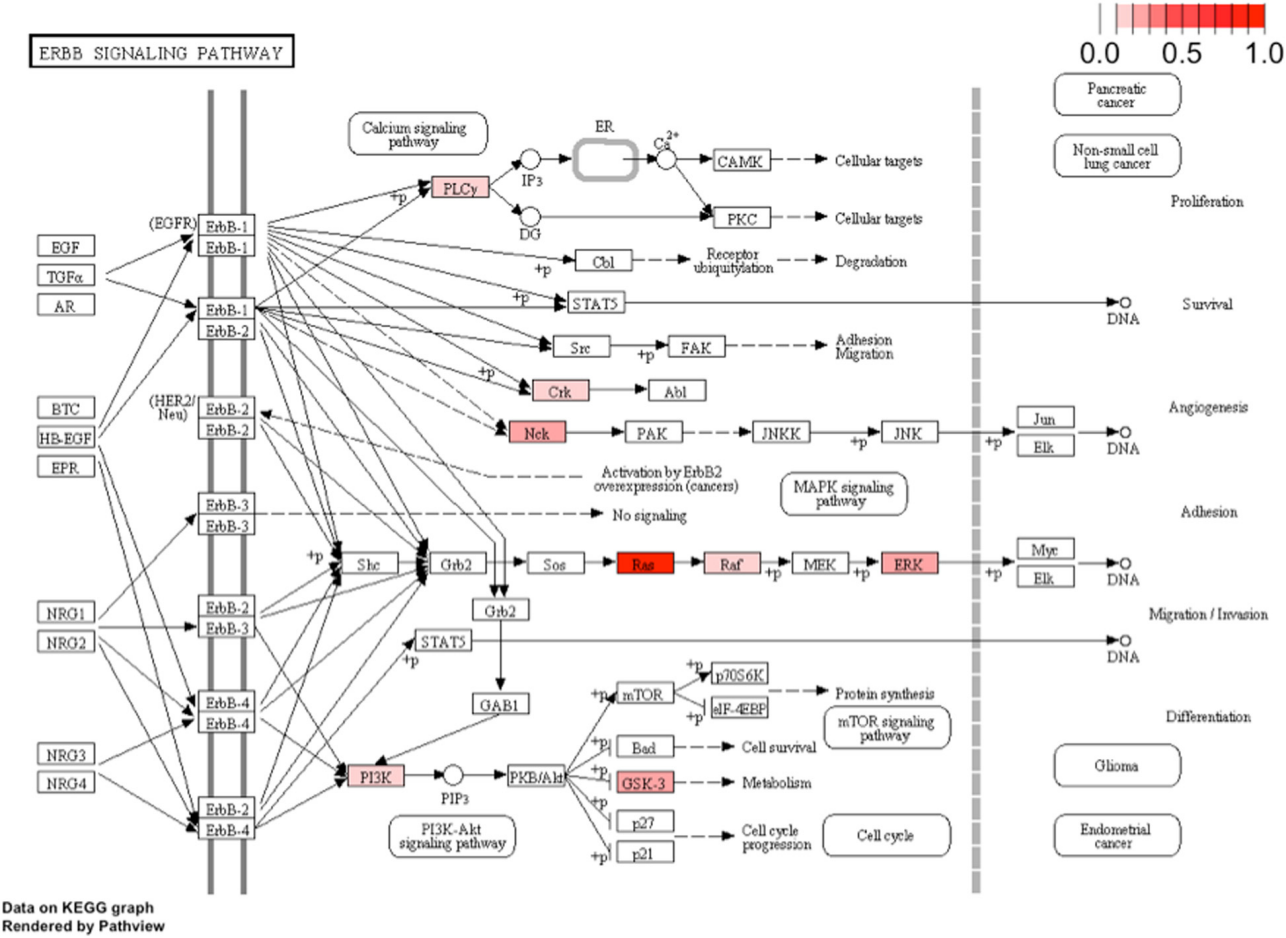
Frequently mutated genes in ErbB signaling pathway. Darker color indicates higher mutation frequencies in mice

Using the CO-GSEA described in the Method Section, we found 95 KEGG pathways that are enriched with CIS-associated genes with permutated *p* value <10^−7^ listed in Table 1 (more details about the disrupted genes in each pathway can be found in Additional file 1). In Table 1, “# of genes” records the number of genes defined in the pathway from KEGG; “# of CIS” (third column) reports the number of CIS genes in the pathway; and “# of mutated cases” (fourth column) records the number of cases that the pathway was disrupted. A histogram of the sizes of each of the KEGG pathways is shown in Additional file 2. In Figs. 1 and 2, we plotted the KEGG diagrams of two pathways that are enriched CIS-associated genes.

**Table 1:** Pathways that are enriched with CIS-associated genes (permuted *p* value <10^−7^)

	KEGG id	Pathway name	# of genes	# of CIS	# of mutated cases
		Cellular Processes			
1	4110	Cell cycle	126	9	101
2	4530	Tight junction	136	10	127
3	4810	Regulation of actin cytoskeleton	217	13	121
4	4510	Focal adhesion	207	13	123
5	4540	Gap junction	87	3	67
6	4520	Adherens junction	74	11	122
		Human Diseases			
7	5164	Influenza A	170	11	117
8	5034	Alcoholism	199	5	85
9	5169	Epstein-Barr virus infection	212	17	120
10	5203	Viral carcinogenesis	229	14	116
11	5160	Hepatitis C	136	7	97
12	5010	Alzheimer’s disease	173	12	121
13	5016	Huntington’s disease	182	14	125
14	5200	Pathways in cancer	323	23	134
15	5211	Renal cell carcinoma	67	6	84
16	5206	MicroRNAs in cancer	270	10	108
17	5152	Tuberculosis	176	11	114
18	5166	HTLV-I infection	277	11	106
19	5100	Bacterial invasion of epithelial cells	77	7	103
20	5412	Arrhythmogenic right ventricular cardiomyopathy (ARVC)	74	7	96
21	5202	Transcriptional misregulation in cancer	178	12	121
22	5133	Pertussis	74	6	84
23	5142	Chagas disease (American trypanosomiasis)	103	7	87
24	5161	Hepatitis B	145	10	107
25	5205	Proteoglycans in cancer	226	11	115
26	5214	Glioma	65	6	95
27	5216	Thyroid cancer	29	3	66
28	5210	Colorectal cancer	64	8	105
29	5212	Pancreatic cancer	66	6	74
30	5213	Endometrial cancer	52	8	111
31	5215	Prostate cancer	89	10	117
32	5218	Melanoma	71	4	86
33	5219	Bladder cancer	38	2	46
34	5220	Chronic myeloid leukemia	73	9	95
35	5221	Acute myeloid leukemia	57	6	82
36	5223	Non-small cell lung cancer	56	5	77
37	5222	Small cell lung cancer	85	7	95
38	5217	Basal cell carcinoma	55	3	65
		Environmental Information Processing			
39	4151	PI3K-Akt signaling pathway	351	22	130
40	4390	Hippo signaling pathway	154	14	123
41	4066	HIF-1 signaling pathway	111	7	91
42	4012	ErbB signaling pathway	87	7	100
43	4014	Ras signaling pathway	228	13	119
44	4310	Wnt signaling pathway	143	16	126
45	4350	TGF-beta signaling pathway	82	11	119
46	4010	MAPK signaling pathway	253	14	118
47	4015	Rap1 signaling pathway	216	14	134
48	4370	VEGF signaling pathway	60	4	68
49	4340	Hedgehog signaling pathway	49	3	61
50	4330	Notch signaling pathway	49	4	68
51	4070	Phosphatidylinositol signaling system	81	4	78
52	4068	FoxO signaling pathway	135	7	101
53	4150	mTOR signaling pathway	61	4	86
		Metabolism			
54	4141	Protein processing in endoplasmic reticulum	169	8	111
55	670	One carbon pool by folate	19	2	45
56	3015	mRNA surveillance pathway	96	8	95
57	4120	Ubiquitin mediated proteolysis	139	14	118
58	00250	Alanine, aspartate and glutamate metabolism	34	2	53
59	3020	RNA polymerase	29	2	50
60	510	N-Glycan biosynthesis	50	3	69
61	3018	RNA degradation	77	5	83
62	310	Lysine degradation	51	6	95
63	512	Mucin type O-Glycan biosynthesis	28	2	46
64	900	Terpenoid backbone biosynthesis	21	2	54
65	563	Glycosylphosphatidylinositol(GPI)-anchor biosynthesis	25	2	46
66	4122	Sulfur relay system	10	3	59
67	4062	Chemokine signaling pathway	196	13	124
68	4722	Neurotrophin signaling pathway	123	12	118
69	4670	Leukocyte transendothelial migration	121	9	106
70	4728	Dopaminergic synapse	133	8	105
71	4270	Vascular smooth muscle contraction	137	8	104
72	4713	Circadian entrainment	98	5	93
73	4723	Retrograde endocannabinoid signaling	103	4	88
74	4724	Glutamatergic synapse	114	5	93
75	4725	Cholinergic synapse	113	5	93
76	4726	Serotonergic synapse	133	5	94
77	4910	Insulin signaling pathway	142	8	95
78	4650	Natural killer cell mediated cytotoxicity	146	5	73
79	4917	Prolactin signaling pathway	74	7	98
80	4611	Platelet activation	131	9	111
81	4912	GnRH signaling pathway	89	5	80
82	4914	Progesterone-mediated oocyte maturation	87	4	68
83	4915	Estrogen signaling pathway	98	6	92
84	4916	Melanogenesis	100	9	111
85	4921	Oxytocin signaling pathway	158	9	108
86	4360	Axon guidance	129	10	117
87	4919	Thyroid hormone signaling pathway	118	11	114
88	4960	Aldosterone-regulated sodium reabsorption	40	2	49
89	4720	Long-term potentiation	66	6	91
90	4660	T cell receptor signaling pathway	105	9	113
91	4662	B cell receptor signaling pathway	73	6	91
92	4730	Long-term depression	61	3	67
93	4664	Fc epsilon RI signaling pathway	70	4	68
94	4666	Fc gamma R-mediated phagocytosis	88	6	86
95	4320	Dorso-ventral axis formation	22	2	60

The genetic screen was designed to discover genes that when altered would cause acceleration of PDA in pancreatic ductal epithelial cells expressing an activated form of the Kras oncogene, *Kras*^*G12D*^. As such it was not surprising that KEGG pathways with the strongest statistical support for CIS associated gene enrichment were many cancer associated pathways. As expected, we found some of the same pathways previously reported and which were expected [7, 8]. An informal prior analysis [7] suggested that *TGF* *β* signaling was enriched in CIS-associated genes and indeed we found that this KEGG pathway is enriched. Similarly, *Rb1/p16Inka4a* pathway was suggested to be recurrently altered by CIS-associated genes [7]. Indeed, we found that the KEGG pathway *CELL CYCLE* was enriched in CIS-associated genes. Many other cancer-associated pathways were enriched in CIS-associated genes including the *RAS*, *PI3K-AKT*, *HIPPO*, *VEGF*, *HEDGEHOG*, *MAPK*, *FOXO1*, and *MTOR* pathways. Moreover, the human disease KEGG pathway *PANCREATIC CANCER* and several other human cancer pathways were enriched in CIS-associated genes.

In addition to these expected KEGG pathways, many involving metabolism have not been strongly linked to pancreatic cancer development or cancer development in general. However, recent studies revealed evidence of metabolic reprogramming to sustain tumor survival in *KRAS*-mutated PDA tumors [9]. For example, KRAS-dependent tumor cells compensated the energy loss through increasing glycolysis, amino acid and lipid biosynthesis [10]. In particular, *TERPENOID BIOSYNTHESIS*, *LYSINE DEGRADATION* and the *SULFUR RELAY SYSTEM* are significantly altered in the SB-accelerated tumor models. To date KRAS remains a poorly druggable target, hence, targeting the downstream metabolic regulation could be effective alternatives in inhibiting tumor growth.

Several organismal systems KEGG pathways were also enriched in CIS-associated genes despite not being strongly linked to pancreatic cancer development. These include *OXYTOCIN SIGNALING*, *CHOLINERGIC SYNAPSE*, and *MELANOGENESIS*. Our recent work helped show that the *AXON GUIDANCE* pathway is enriched for CIS-associated genes, a result which led to the discovery that these genes and the pathways they participate in are altered in human PDA [8]. This result was reproduced in this current analysis. Thus, it is clear that the broadened definition of CIS allows for the identification of many known and novel candidate cancer pathways. These data suggest many new hypotheses to be tested in PDA development.

### Analysis of individual tumor reveals significant co-altered pathways

**Fig. 3:**
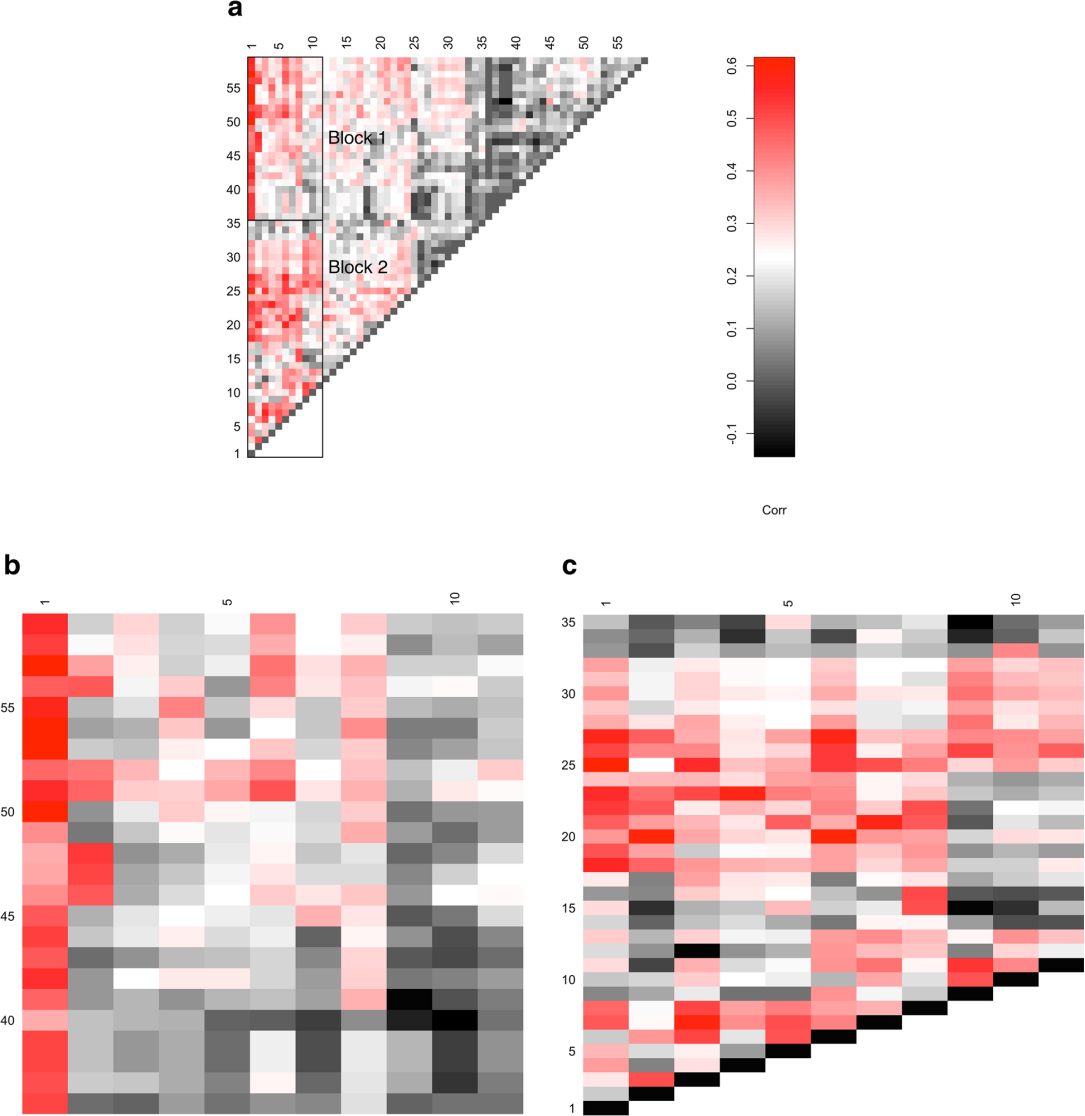
Heat map of correlation between pair of pathways. Legend indicates strength of correlation coefficient (*red*: high correlation; *black*: weak correlation). **a** Heat map of correlation between all pairs of pathways. **b** Zoom in of block 1 shown in panel **a**. **c** Zoom in of block 2 shown in panel **a**. Pathway names from left to right (bottom to top): 1: Ubiquitin mediated proteolysis, 2: One carbon pool by folate, 3: Wnt signaling pathway, 4: Cell cycle, 5: Hippo signaling pathway, 6: Protein processing in endoplasmic reticulum, 7: MAPK signaling pathway, 8: Lysine degradation, 9: RNA polymerase, 10: N-Glycan biosynthesis, 11: mRNA surveillance pathway, 12: Hedgehog signaling pathway, 13: Dopaminergic synapse, 14: GPI-anchor, 15: RNA degradation, 16: Terpenoid backbone biosynthesis, 17: Mucin type O-Glycan biosynthesis, 18: Rap1 signaling pathway, 19: Adherens junction, 20: Leukocyte transendothelial migration, 21: TGF-beta signaling pathway, 22: Axon guidance, 23: MicroRNAs in cancer, 24: Tight junction, 25: PI3K-Akt signaling pathway, 26: Ras signaling pathway, 27: Chemokine signaling pathway, 28: Serotonergic synapse, 29: Glutamatergic synapse, 30: Cholinergic synapse, 31: Retrograde endocannabinoid signaling, 32: Circadian entrainment, 33: Sulfur relay system, 34: Notch signaling pathway, 35: Alanine, aspartate and glutamate metabolism, 36: Aldosterone-regulated sodium reabsorption, 37: Natural killer cell mediated cytotoxicity, 38: VEGF signaling pathway, 39: Fc epsilon RI signaling pathway, 40: Gap junction, 41: Melanogenesis, 42: FoxO signaling pathway, 43: HIF-1 signaling pathway, 44: Fc gamma R-mediated phagocytosis, 45: Estrogen signaling pathway, 46: Platelet activation, 47: Oxytocin signaling pathway, 48: Vascular smooth muscle contraction, 49: PIP, 50: mTOR signaling pathway, 51: Focal adhesion, 52: Regulation of actin cytoskeleton, 53: Insulin signaling pathway, 54: Thyroid hormone signaling pathway, 55: ErbB signaling pathway, 56: T cell receptor signaling pathway, 57: Neurotrophin signaling pathway, 58: B cell receptor signaling pathway, 59: Prolactin signaling pathway

We and others have published results of SB screens in which we found that individual CIS tended to be co-mutated by transposon insertion more than expected by chance (e.g. [11]). We wondered whether an analysis of individual tumors would reveal that specific pathways would be co-altered in this same manner. Figure 3 shows a heat map of adjusted correlation between pair of pathways, which are co-altered by transposon insertions within/near genes in those pathways. We observed that there are two major clusters of strongly co-altered pathways. Within these clusters certain specific pathways show strong associations, being altered by transposon insertion in the same tumors more often than would be expected by chance. These data provide the basis for developing specific hypotheses about pathways that interact to cause cancer. Thus, alterations of one pathway may allow the other pathway to exert its full oncogenic effects.

A careful analysis of some of the associations reveals pairs of pathways that might be predicted to interact based on what is known about their functions and regulation already. For example, block 1, labeled in Fig. 3, contains strong associations between the ubiquitin processing pathway and several pathways including *ErbB*, *Insulin* and *mTOR* signaling. It is known that cell signaling pathways that transmit signals from the extracellular space into the cell cytoplasm and nucleus are regulated by the abundance and stability of certain proteins. In many cases, the stability of these proteins is regulated by ubiquitination and degradation by the proteosome. Well known examples, include *NF* *κB* and *Wnt/ β-catenin* signaling pathways. Work shows that members of the ErbB family of receptors are downregulated by ubiquitination involving the E3 ubiquitin ligase Cbl [12]. Ubiquitination also regulates *Akt-mTOR* signaling in multiple myeloma [13] and *Akt-mTOR* is activated by *insulinsignaling* [14].

Block 2, labeled in Fig. 3 contains several other intriguing pathway-pathway associations. For example, we see a strong association between cell cycle control and miRNAs known to be involved in cancer. Indeed, there are several well studied examples of miRNAs that regulate the mRNA transcripts of cell cycle regulators such as MYC [15], RB1 [16] and CCND1 [17]. Also in block 2, we see evidence for *TGF β* pathway and *MAPK* pathway co-dysregulation. Abundant evidence for crosstalk between these pathways has been published [18, 19]. Thus, it is entirely plausible that co-alteration between these pathways is specifically selected for during PDA progression. Specific hypotheses can, or have been, tested in the laboratory. For example, MAPK activation, via expression of the Kras^G12D^ oncogene, cooperates strongly with Smad4 inactivation, which alters/inactivates *TGF β* signaling, in a mouse model of PDA [20]. This functionally confirms the observation from the analyses done here. We can thus predict, that many other pathway-pathway associations observed in Fig. 3 can be functionally validated. More speculative, but of tremendous therapeutic significance, is the idea that targeting one pathway of a pathway-pathway pair observed in Fig. 3 would alter the ability of the second pathway to exert its oncogenic effects. Indeed, co-targeting both of such pairs of altered pathways may be the most effective way to treat individual cases of PDA. These ideas wait functional testing in the laboratory using model systems.

### Association of CIS-associated genes and enriched pathways

**Fig. 4:**
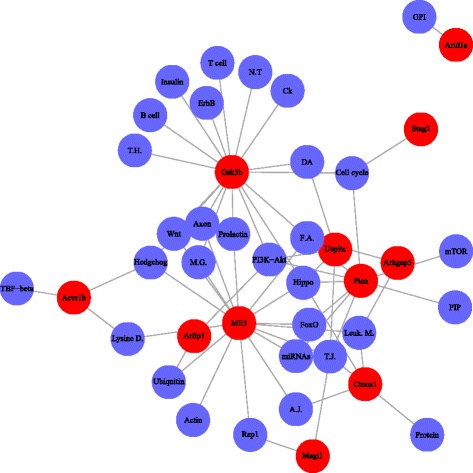
Associations between CIS and enriched pathways. Red nodes represent CIS associated genes, and blue nodes indicate pathways. Abbreviations: miRNAs: MicroRNAs in cancer, GPI: Glycosylphosphatidylinositol-anchor biosynthesis, T.J.: Tight junction, Hedgehog: Hedgehog signaling pathway, PIP: Phosphatidylinositol signaling system, B cell: B cell receptor signaling pathway, Leuk. M.: Leukocyte transendothelial migration, DA: Dopaminergic synapse, Axon: Axon guidance, Ubiquitin: Ubiquitin mediated proteolysis, F.A: Focal adhesion, M.G.: Melanogenesis, Lysine D.: Lysine degradation, Protein: Protein processing in endoplasmic reticulum, T cell: T cell receptor signaling pathway, T.H.: Thyroid hormone signaling pathway, N.T: Neurotrophin signaling pathway, A.J.: Adherens junction, Actin: Regulation of actin cytoskeleton, Ck: Chemokine signaling pathway

Several of the most commonly altered genes in the PDA screen (i.e. the top ranked CIS-associated genes) have little published functional data. We speculate that by finding which pathways they most often interact with, something could be learned about their function in general and in PDA development. The associations between the top ranked CIS and enriched pathways are shown in Fig. 4. In Fig. 4, several CIS-associated genes such as *Stag2*, *Arhgap5*, *Usp9x*, *Magi1*, *Arid1a* have few connections to enriched pathways then other CIS-associated genes. In Additional file 3, we listed these connections and corresponding estimates from regression model, *p* values and FDR. For example, in Additional file 1, *Usp9x* is associated with *PI3K-AKT signaling* pathway, *DOPAMINERGICSYNAPSE*, *HIPPO signaling*, and *TIGHT JUNCTION* pathways. Thus, it seems likely that *Usp9x* mutation or down regulation has to cooperate with alterations in these other pathways in order for PDA to develop. The CIS-associated genes that also demonstrated association with the these *Usp9x*-associated pathways are *Gsk3b*, *Ctnna1*, *Mll5*, *Pten*, *Arfip1*, *Magil*.

## Conclusion

In this work, we demonstrate the non-random enrichment of CIS-associated genes from a transposon-based screen for PDA into certain KEGG signaling pathways, disease states and biological processes.

## Methods

To assess whether a pathway harbors more CIS-associated genes than expected by chance, we use CO-GSEA approach. For each tumor sample, we considered a pathway is altered (coded: 1) if at least 1 gene in the pathway was mutated; coded zero if it’s not. A score for each pathway was calculated to be the number of tumors in which the pathway is altered. We assessed whether the score of a pathway was statistically significant through random permutation. For example, if a mouse tumor contains 100 mutations, we randomly assigned the 100 mutations to 100 different genes. A score for each pathway can be obtained by counting the number of altered tumor samples after the permutation. We repeated the permutation 10^7^ times to obtain the distribution of score under the null for each pathway and calculated a *p* value based on the permuted null distribution. A similar approach was also applied in mutation analysis of human tumor samples in [6].

The ability to detect a significant pathway using the CO-GSEA approach depends on the background mutation rate and the size of the pathway under consideration. The relationship between the number of total cases, and the expected score of a given pathway under random permutation can be described as: $N- \Sigma _{i=1}^{N}\tiny {\frac {\left (\! \begin {array}{c} G-n_{i} \\ P_{s} \end {array} \!\right)} {\left (\! \begin {array}{c} G\\ P_{s} \end {array} \!\right)}}$, where *N* is the total number of cases; G is the number of genes considered in the pathway analysis; *n*_*i*_ is the number of events in sample *i* and *P*_*s*_ is the number of genes in the pathway [6].

### Analysis of co-altered pathways

To investigate whether a pair of pathways was co-altered in a significant manner, we remove the CIS-associated genes that are present in both pathways, and for each sample, we calculated the mutation frequency in each pathway using the remaining non-overlapping CIS as: $\frac {\text {\# of mutations in sample } i\text { in the pathway}}{\text {\# of non-overlapping CIS in the pathway}}$. For each pair of pathways, Pearson correlations were calculated to present the correlation between pathways characterized by non-overlapping CIS using the mutation counts.

### Association between top CIS-associated genes and enriched pathways

Among the top 20 CIS-associated genes previously reported [7], 12 of them listed do not map to any KEGG pathways. We conducted association analysis between the top 20 CIS-associated genes and the enriched KEGG pathway using quasi-Poisson regression models with over-dispersion. For each CIS-associated gene, we examined whether mutation status of the CIS-associated gene (code 1 if mutated; 0 otherwise) is associated with higher mutation counts (the number of altered CIS-associated genes) for a pathway under consideration. We reported the CIS and pathways associations with FDR < 0.001.

## Additional files

Additional file 1 Significant CIS enriched pathways and disrupted genes. Number in the parenthesis represents # of cases with the gene mutated. (CSV 13.4 kb)

Additional file 2 Histogram of gene set sizes. (PNG 25.3 kb)

Additional file 3 CIS and pathways association table. (TXT 3.87 kb)
