# Supplementary figures and images for: Case-oriented pathways analysis in pancreatic adenocarcinoma using data from a sleeping beauty transposon mutagenesis screen
Source: BMC Med Genomics. 2016 Apr 1;9:16. doi: 10.1186/s12920-016-0176-7 (PMC4818883; doi:10.1186/s12920-016-0176-7)

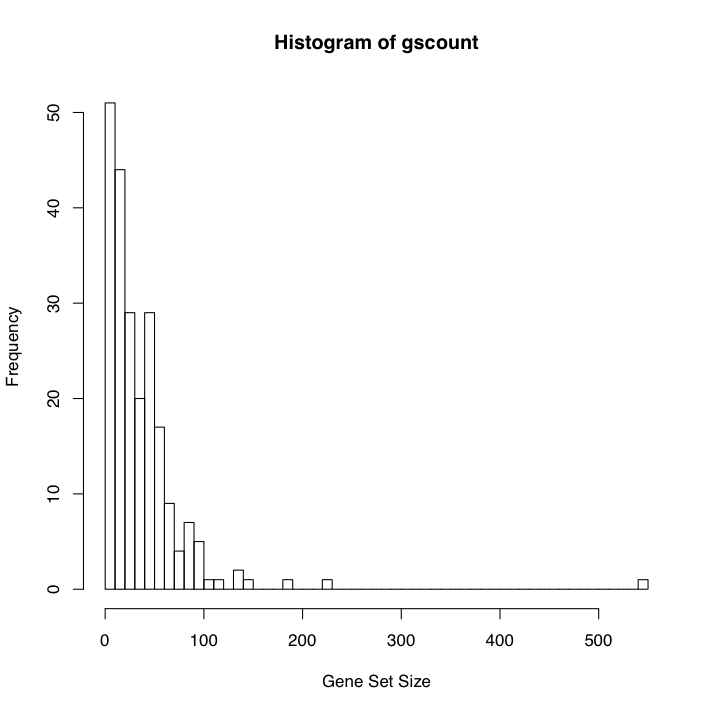

Supplement: Supplementary file 2 — Histogram of gene set sizes. (PNG 25.3 kb) [file 12920_2016_176_MOESM2_ESM.png]
